# Integrated Transcriptome and WGCNA Analyses Reveal Candidate Regulatory Networks Associated with Shell Hardening in *Macadamia integrifolia*

**DOI:** 10.3390/plants15162442

**Published:** 2026-08-11

**Authors:** Qingyi Long, Yang Li, Jianjian Geng, Lidan Gong, Chao Wu, Jing Ma, Tingyu Li, Nanhua Zi, Xinghao Tu, Liang Tao

**Affiliations:** 1 Yunnan Institute of Tropical Crops, Jinghong 666100, China; lqingyi@163.com (Q.L.);; 2 Key Laboratory of Tropical Fruit Biology, Ministry of Agriculture & Rural Affairs, South Subtropical Crops Research Institute of Chinese Academy of Tropical Agricultural Sciences, Zhanjiang 524091, China

**Keywords:** *Macadamia integrifolia*, shell, pericarp, secondary cell wall, transcriptome, RNA-seq

## Abstract

To characterize the transcriptomic dynamics underlying shell hardening in *Macadamia integrifolia*, RNA sequencing was performed on pericarp and shell tissues collected at three key developmental stages. A total of 18 libraries were generated, yielding 125.19 Gb of clean data. Gene Ontology (GO) and Kyoto Encyclopedia of Genes and Genomes (KEGG) analyses showed that husk-associated DEGs were mainly enriched in photosynthesis-related functions, whereas shell-associated DEGs were enriched in phenylpropanoid biosynthesis, secondary metabolism, and cell wall organization. Weighted gene co-expression network analysis (WGCNA) identified module M8 as being strongly associated with the middle and late stages of shell development. Genes in this module were mainly enriched in phenylpropanoid metabolism, redox-related processes, and extracellular region functions. The hub genes within M8 included those encoding a glutaredoxin family protein, a WNK-type protein kinase, a tRNA methyltransferase-related protein, an EFR3 membrane-associated protein, a pectin modification-related protein, and a WAT1-related protein. Collectively, these results reveal clear tissue-specific transcriptomic patterns during pericarp and shell development and identify candidate co-expression networks that may contribute to shell hardening.

## 1. Introduction

*Macadamia* spp. is an economically important woody oil crop that has attracted increasing attention because of its high nutritional value and broad potential for industrial utilization. Native to Australia, *M. integrifolia* is now widely cultivated in Australia, China, South Africa, Kenya, the United States (Hawaii), and several other tropical and subtropical regions worldwide. Its kernels are rich in monounsaturated fatty acids, proteins, dietary fiber, vitamins, minerals, and antioxidant compounds, making *M. integrifolia* one of the highest-value edible nuts in the global market. Owing to its high nutritional quality and broad applications in the food, cosmetic, and health industries, *M. integrifolia* has become a valuable commercial crop in many producing countries. Consequently, improving fruit quality and understanding the molecular mechanisms underlying shell development are of considerable importance for breeding and industrial production [1,2,3]. In *M. integrifolia* fruit, the shell serves as the main protective tissue enclosing the seed and is essential for maintaining structural integrity and mechanical strength. Its physical properties directly influence fruit harvesting, transportation, storage, and shelling efficiency [2,3]. Shell hardening is a critical developmental process that is generally accompanied by secondary cell wall thickening and lignin deposition, both of which are central to the formation of nut fruit structure [4]. Although previous studies have examined shell metabolism and transcriptomic variation, as well as pericarp formation and metabolic changes during pericarp development in *M. integrifolia*, integrated analyses of coordinated pericarp and shell development remain scarce. This limitation has hindered a more comprehensive understanding of the molecular mechanisms underlying shell hardening [4].

Previous studies have demonstrated that the formation of hardened shell tissues in nuts and drupes is closely associated with phenylpropanoid metabolism and its downstream lignin biosynthetic pathway, and that this process often displays pronounced stage-specific transcriptional patterns during fruit development [5,6,7]. As fruit development progresses, functional differentiation among tissues becomes increasingly evident. Outer tissues such as the pericarp generally retain photosynthetic capacity and primary metabolic activity, whereas mechanically protective tissues are more strongly engaged in cell wall assembly and secondary metabolism [8]. In *M. integrifolia*, however, comprehensive evidence remains lacking regarding the transcriptional division of labor between the pericarp and shell throughout development and their respective contributions to shell hardening. Although previous studies have provided insights into phenolic and flavonoid metabolism in the shell or endocarp, as well as the metabolic characteristics of pericarp development [9,10], several important questions remain unresolved. In particular, the critical developmental windows during which transcriptomic reprogramming occurs during shell hardening, the patterns of tissue-specific expression divergence, and the coordinated regulation of core metabolic pathways have yet to be systematically elucidated using multi-stage transcriptomic data.

Recent years have witnessed substantial progress in understanding structural formation in nut fruits [4,11,12]. Studies in species such as walnut (*Juglans regia*) and iron walnut (*Juglans sigillata*) have shown that genes involved in phenylpropanoid metabolism and cell wall biogenesis are markedly upregulated during shell formation, with expression typically peaking at intermediate developmental stages. Lignin deposition has likewise been recognized as a major determinant of structural reinforcement in fruits such as walnut and winter jujube [5,6,13]. In addition, multi-omics studies have highlighted the coordinated involvement of hormone signaling, redox regulation, and secondary metabolism during fruit development [9,10,12,14]. Together, these findings have advanced the molecular understanding of fruit structural formation from multiple perspectives [9,10]. However, most previous studies have focused on individual tissues or specific developmental stages, and systematic comparisons of coordinated pericarp and shell development remain limited [5,6,9,10]. Although differential expression analysis has been widely applied, the integration of transcriptional changes at the co-expression network level is still relatively limited, which restricts a more comprehensive understanding of the regulatory mechanisms underlying complex developmental traits [14,15,16]. In *M. integrifolia*, existing research has mainly focused on genomic resources and metabolic variation, whereas the transcriptional regulatory networks associated with shell hardening remain largely unexplored [9]. Therefore, integrating multi-stage transcriptomic data with co-expression network analysis is necessary to clarify the expression characteristics and regulatory relationships underlying shell hardening [14,15,16].

To address these knowledge gaps, the present study aimed to systematically characterize the transcriptomic dynamics underlying *M. integrifolia* shell hardening by integrating comparative transcriptome analysis with weighted gene co-expression network analysis (WGCNA). Specifically, we sought to identify tissue-specific expression patterns, key developmental stages, biological pathways, and candidate hub genes associated with shell development. ranscriptome sequencing of green pericarp (husk) and shell tissues collected at three developmental stages. Sequencing quality and global expression patterns were first evaluated to characterize the overall transcriptomic landscape. Differential expression analysis was then conducted to identify tissue- and stage-specific transcriptional variation, followed by Gene Ontology (GO) and Kyoto Encyclopedia of Genes and Genomes (KEGG) enrichment analyses to determine the major biological processes and metabolic pathways involved. WGCNA was subsequently employed to construct co-expression networks, identify key functional modules, and screen hub genes associated with shell hardening. These findings provide new insights into the molecular mechanisms underlying shell structural formation in *M. integrifolia*.

## 2. Materials and Methods

### 2.1. Plant Materials and Sample Preparation

The materials were collected from the main cultivated *M. integrifolia* variety (HAES900), at the Ministry of Agriculture Jinghong Macadamia Germplasm Resource Nursery, Xishuangbanna, Yunnan Province, China (22.013188° N, 100.776066° E). The orchard was managed under conventional intensive cultivation, with a row spacing of 5 m and a plant spacing of 7 m. All sampled trees were 14 years old, vigorous, free of visible pests and diseases, and exhibited uniform growth.

According to fruit developmental progression, samples were collected at 30, 50, and 80 days after flowering (DAF). The corresponding pericarp (husk) samples were designated H-1, H-2, and H-3, and the corresponding shell samples were designated S-1, S-2, and S-3, respectively. For each tissue at each developmental stage, three biological replicates were collected, with each replicate obtained from a different individual tree, giving a total of 18 samples. At each sampling time point, fruits of normal size and consistent growth were randomly harvested from the sun-exposed side of the middle canopy. The pericarp and shell tissues were immediately separated on ice. The kernel and membranous inner seed coat were carefully removed to avoid cross-contamination between tissues. The target tissues were then cut into pieces of approximately 0.5–1.0 cm, immediately frozen in liquid nitrogen, and stored at −80 °C until RNA extraction and transcriptome sequencing.

### 2.2. Total RNA Extraction and Quality Assessment

Both pericarp and shell tissues are rich in polyphenols, polysaccharides, and cell wall components. Therefore, all sample processing steps were carried out under low-temperature conditions. Approximately 100 mg of frozen tissue from each sample was ground into a fine powder in liquid nitrogen, and an appropriate amount of polyvinylpyrrolidone (PVP) was added during grinding to reduce interference from polyphenolic compounds. Total RNA was extracted using the RNAprep Pure Plant Plus Kit (Polysaccharides & Polyphenolics-rich; Catalog No. DP441, TIANGEN Biotech, Beijing, China) according to the manufacturer’s instructions, including lysis, purification, and on-column DNase I digestion to remove residual genomic DNA.

RNA quality was evaluated using a NanoDrop spectrophotometer (Thermo Fisher Scientific, Wilmington, DE, USA) to assess purity (OD260/OD280 and OD260/OD230), a Qubit fluorometer (Thermo Fisher Scientific, Waltham, MA, USA) to determine RNA concentration, and 1% agarose gel electrophoresis to examine RNA integrity. The RNA integrity number (RIN) was further determined using an Agilent 2100 Bioanalyzer (Agilent Technologies, Santa Clara, CA, USA). Only RNA samples meeting the following criteria were used for subsequent library construction: RIN ≥ 7.0, OD260/OD280 between 1.8 and 2.2, and OD260/OD230 > 2.0 [17,18].

### 2.3. Library Construction and Illumina Sequencing

For each sample, 1–2 μg of high-quality total RNA was used for strand-specific mRNA library construction. Poly(A)+ mRNA was enriched using oligo(dT)-coated magnetic beads and then fragmented into suitable sizes in the presence of divalent cations. First-strand cDNA was synthesized using random primers, and dUTP was incorporated during second-strand synthesis to maintain strand specificity. The resulting cDNA was then subjected to end repair, A-tailing, adapter ligation, fragment size selection, and PCR amplification to generate sequencing libraries with an insert size of approximately 300 bp [19,20]. Library quality was assessed using an Agilent 2100 Bioanalyzer and a Qubit fluorometer, after which paired-end sequencing (PE150) was performed on the Illumina NovaSeq 6000 platform (Illumina, San Diego, CA, USA).

### 2.4. Sequencing Data Quality Control and Filtering

After raw sequencing data were generated in FASTQ format, fastp v0.23.2 was used for adapter trimming, low-quality read filtering, and quality assessment. During this process, reads containing adapter contamination, excessive ambiguous bases (N), or low-quality bases were removed to obtain clean reads. The number of clean reads, Q30 values, and GC content were then calculated for each sample to evaluate sequencing quality [21,22].

### 2.5. Read Alignment and Expression Quantification

The quality-filtered clean reads were aligned to the *Macadamia integrifolia* reference genome assembly SCU_Mint_v3, which was obtained from the NCBI RefSeq database under accession number GCF_013358625.1. The genome sequence file and structural annotation file used in this study were GCF_013358625.1_SCU_Mint_v3_genomic.fna and GCF_013358625.1_SCU_Mint_v3_genomic.gff, respectively. Read mapping was performed using HISAT2 v2.1.0 with default parameters [23]. Based on the reference genome annotation file, featureCounts v2.0.1 was used to quantify the number of reads mapped to each gene, thereby generating a gene-level raw count matrix for subsequent differential expression analysis [21]. In addition, the overall mapping rate, uniquely mapped read rate, and the distribution of reads across exon, intron, and intergenic regions were calculated for each sample based on the alignment results and genome annotation file. These metrics were used to comprehensively assess the suitability of the reference genome for transcriptome analysis and the completeness of genome annotation.

To support downstream sample correlation analysis, principal component analysis (PCA), and co-expression network analysis, the raw count matrix was further normalized. Correlation analysis and PCA were performed using the variance-stabilizing transformation (VST) matrix generated by DESeq2 v1.38.3, whereas WGCNA was conducted using the normalized expression matrix for network construction [21,24].

### 2.6. Identification of Differentially Expressed Genes

Differentially expressed genes (DEGs) were identified using the DESeq2 package in the R environment. DESeq2 models RNA-seq raw count data using a negative binomial distribution and improves the robustness of differential expression analysis through dispersion estimation and shrinkage [25]. The gene-level raw count matrix generated by featureCounts was used as input for pairwise comparisons between pericarp and shell tissues at the same developmental stage, as well as between different developmental stages within the same tissue. Statistical significance was assessed using the Wald test, and *p* values were adjusted for multiple testing using the Benjamini–Hochberg method. Genes with an FDR ≤ 0.05 and an absolute log2 fold change (|log2FoldChange|) ≥ 1 were considered significantly differentially expressed [25,26].

### 2.7. Functional Annotation and Enrichment Analysis

Functional annotation of the complete gene set and target gene sets was performed based on the *M. integrifolia* reference genome annotation in combination with the NR, Swiss-Prot, Pfam, Gene Ontology (GO), and Kyoto Encyclopedia of Genes and Genomes (KEGG) databases. GO classification and enrichment analyses, together with KEGG pathway enrichment analyses, were conducted separately for the identified DEGs and for genes within key WGCNA modules. The complete set of expressed genes detected across all RNA-seq samples was used as the background gene set for both GO and KEGG enrichment analyses. Statistical significance was evaluated using the hypergeometric test or Fisher’s exact test, *p* values were adjusted for multiple testing using the Benjamini–Hochberg (BH) procedure to control the false discovery rate (FDR), and GO and KEGG terms with an adjusted *p* value (FDR) < 0.05 were considered significantly enriched [27,28].

### 2.8. Weighted Gene Co-Expression Network Analysis (WGCNA)

To identify gene sets showing coordinated expression changes during shell development and to detect co-expression modules significantly associated with the middle and late stages of shell development, a weighted gene co-expression network was constructed using a normalized FPKM expression matrix. Before network construction, genes with low expression levels (FPKM < 1 in more than 50% of samples) were removed, and genes with low expression variability were further filtered using the varFilter function implemented in the R package genefilter (v1.80.3). Specifically, genes ranked within the top 50% based on expression variance across samples were retained for subsequent WGCNA. Network construction was performed in R (v4.2.2) using the WGCNA package. The soft-thresholding power was selected according to the scale-free topology criterion, and a soft-thresholding power of β = 6 was used for subsequent network construction. Pearson correlation coefficients between genes were first transformed into an adjacency matrix, after which a topological overlap matrix (TOM) was calculated [14]. Co-expression modules were then identified by hierarchical clustering combined with the Dynamic Tree Cut algorithm using a deepSplit value of 2 and a minimum module size of 30 genes, and different modules were distinguished by different colors [15,16]. Module eigengenes were subsequently calculated and correlated with sample traits, including tissue type, developmental stage, and days after flowering (DAF), using Pearson correlation analysis. Module-trait relationships with *p* < 0.05 were considered statistically significant.

After the module eigengene was calculated for each module, correlation analyses were performed between module eigengenes and sample traits, including tissue type (pericarp/shell), developmental stage, and days after flowering (DAF), to identify modules significantly associated with shell development. Candidate hub genes within each module were screened on the basis of intramodular connectivity (kWithin), with genes showing higher kWithin values considered as highly connected candidate hub genes within the corresponding modules, and their potential functions were further interpreted in combination with GO and KEGG annotation results [15,16].

### 2.9. Quantitative Real-Time PCR Validation

Eleven genes (Supplementary Text S1) were selected for quantitative real-time PCR (qRT-PCR) validation of the RNA-seq results, and their primer sequences and amplification characteristics are provided in Table S1. Total RNA extracted from the same biological samples used for RNA-seq was reverse-transcribed using the PrimeScript™ RT Reagent Kit with gDNA Eraser (Takara Bio, Dalian, China). Gene-specific primers were designed using Primer3, with expected amplicon sizes of 80–200 bp. The actin gene (*ACT*) was used as the internal reference gene because of its reported expression stability in *Macadamia integrifolia* [29]. The *ACT* primer sequences were 5′-GAGGAGAGGATCTGTCGTAAA-3′ (forward) and 5′-GATAACAAGGAGAGGCCAAAG-3′ (reverse). qRT-PCR was performed using TB Green^®^ Premix Ex Taq™ II (Takara Bio Inc., Kusatsu, Shiga, Japan) on a QuantStudio™ 5 Real-Time PCR System (Applied Biosystems, Foster City, CA, USA). Each 20 μL reaction contained 10 μL of 2× premix, 0.8 μL each of the forward and reverse primers, 2 μL of diluted cDNA, and 6.4 μL of nuclease-free water. The thermal cycling conditions were 95 °C for 30 s, followed by 40 cycles of 95 °C for 5 s and 60 °C for 30 s. Amplification specificity was confirmed by melting-curve analysis and no-template controls. Primer efficiencies were evaluated using five-fold serial dilutions of pooled cDNA. In accordance with the MIQE 2.0 guidelines [30], only primer pairs with amplification efficiencies of 90–110% and $R^{2}>0.99$ were used. Three biological replicates, each comprising three technical replicates, were analyzed. Relative expression levels were calculated using the $2^{-\Delta \Delta Cq}$ method, with *ACT* as the internal reference gene and the S1 shell sample as the calibrator [31].

## 3. Results

### 3.1. Sequencing Data Quality and Alignment Statistics

**Figure 1:**
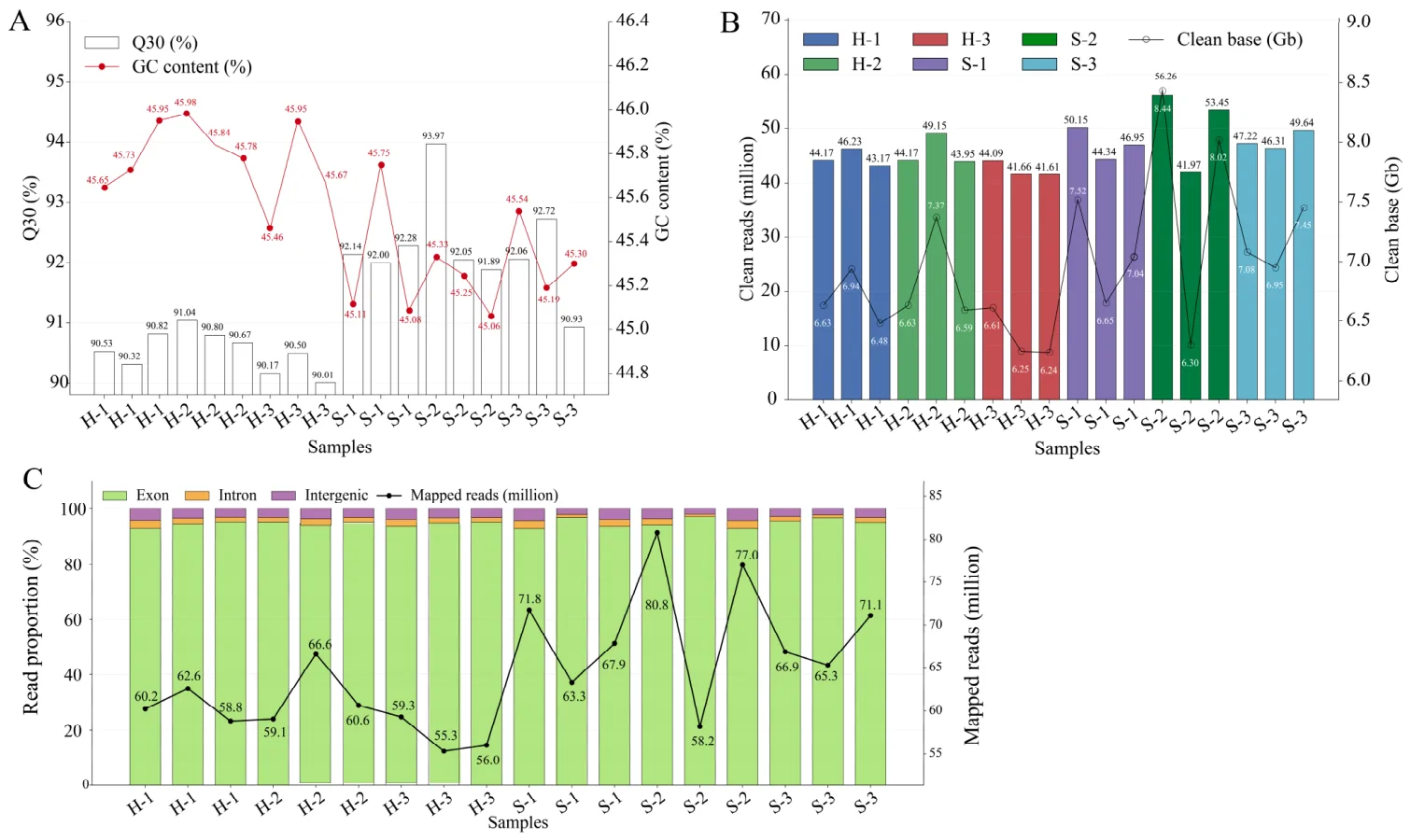
Sequencing data quality and alignment statistics. (**A**) Distribution of Q30 proportion and GC content across samples; (**B**) Statistics of clean reads and clean bases for each sample; (**C**) Distribution proportions of reads in exon, intron, and intergenic regions for each sample, with the line graph indicating the number of mapped reads.

To assess the quality and alignment performance of the transcriptome sequencing data, sequencing output, base quality, GC content, and read mapping statistics were analyzed for all 18 samples (Figure 1; Table 1). The Q30 values ranged from 90.01% to 93.97%, with an average of 91.38%, while GC content ranged from 45.06% to 45.98%, with an average of 45.53% (Figure 1A; Table 1). Among the samples, the highest Q30 value was 93.97% and the lowest was 90.01%. In contrast, GC content varied only slightly across samples.

Read mapping against the *M. integrifolia* SCU_Mint_v3 reference genome showed that the overall alignment rate ranged from 88.50% to 93.43%, with an average of 91.49%, whereas the unique alignment rate ranged from 85.30% to 90.67%, averaging 88.38% (Table 1). In total, 8.34 × 10^8^ clean reads were generated from the 18 libraries, corresponding to 125.19 Gb of clean bases. On average, each sample yielded 46.36 × 10^6^ clean reads and 6.96 Gb of clean bases (Figure 1B; Table 1). Among all samples, the S-2 stage showed the highest number of clean reads (56.26 × 10^6^) and clean bases (8.44 Gb), whereas the H-3 stage showed the lowest values, with 41.61 × 10^6^ clean reads and 6.24 Gb of clean bases.

Analysis of read distribution across genomic regions showed that exonic regions accounted for the majority of mapped reads in all 18 samples, ranging from 91.83% to 97.09%. By contrast, intronic regions accounted for 0.89–3.45% of reads, and intergenic regions accounted for 2.01–4.73% (Figure 1C). Overall, the distribution patterns were highly consistent among samples, with exonic regions consistently representing the predominant proportion of reads.

### 3.2. Global Transcriptomic Expression Patterns of Husk and Shell Samples

**Figure 2:**
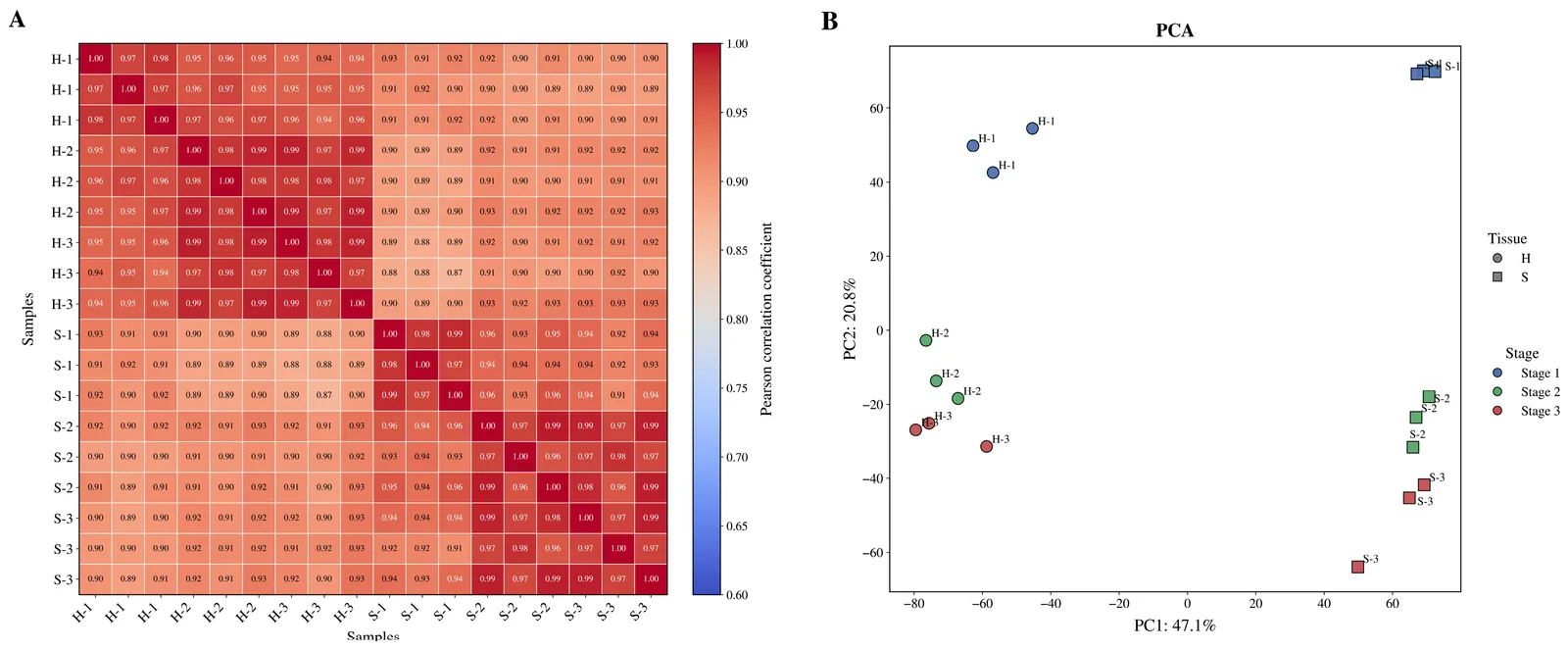
Global transcriptomic patterns among samples. (**A**) Heatmap of Pearson correlation coefficients among samples, with color intensity indicating the strength of correlation. (**B**) Principal component analysis (PCA) plot showing the distribution of samples, in which different colors or symbols represent husk and shell tissues at different developmental stages. Each developmental stage included three biological replicates (*n* = 3). Pearson correlation analysis and PCA were performed using the variance-stabilizing transformation (VST)-normalized expression matrix generated by DESeq2.

To compare global transcriptomic differences between husk and shell samples, Pearson correlation analysis and principal component analysis (PCA) were performed on all 18 samples. The Pearson correlation heatmap showed that biological replicates from the same tissue at the same developmental stage generally exhibited high correlation coefficients (Figure 2A). Specifically, correlation coefficients among replicates at the H-2, H-3, S-1, S-2, and S-3 stages were mostly between 0.91 and 0.99. Correlations among H-1 replicates were slightly lower, but still ranged from 0.94 to 0.95. In general, correlations between different tissues were lower than those within the same tissue. Notably, correlations between middle-to-late husk samples (H-2 and H-3) and early shell samples (S-1) were relatively low, with the lowest values ranging from 0.52 to 0.59. Overall, the samples formed relatively distinct clusters, primarily according to tissue type (husk vs. shell) and developmental stage.

PCA further revealed clear separation among samples along the first two principal components (Figure 2B). PC1 explained 47.1% of the total variance, whereas PC2 explained 20.8%. Husk samples were mainly distributed on the negative side of PC1, whereas shell samples were primarily located on the positive side, indicating marked differences in global expression profiles between the two tissue types. Along the PC2 axis, husk samples were sequentially arranged according to developmental stage from H-1 to H-2 to H-3. Similarly, shell samples showed progressive separation from S-1 to S-2 to S-3, indicating clear transcriptomic divergence across developmental stages. In addition, biological replicates at each stage clustered closely together, with limited dispersion among replicates. Taken together, the Pearson correlation and PCA results demonstrated strong consistency among biological replicates, clear transcriptomic separation between husk and shell tissues, and distinct expression differences among developmental stages.

### 3.3. Differential Gene Expression Profiles During Husk and Shell Development

**Figure 3:**
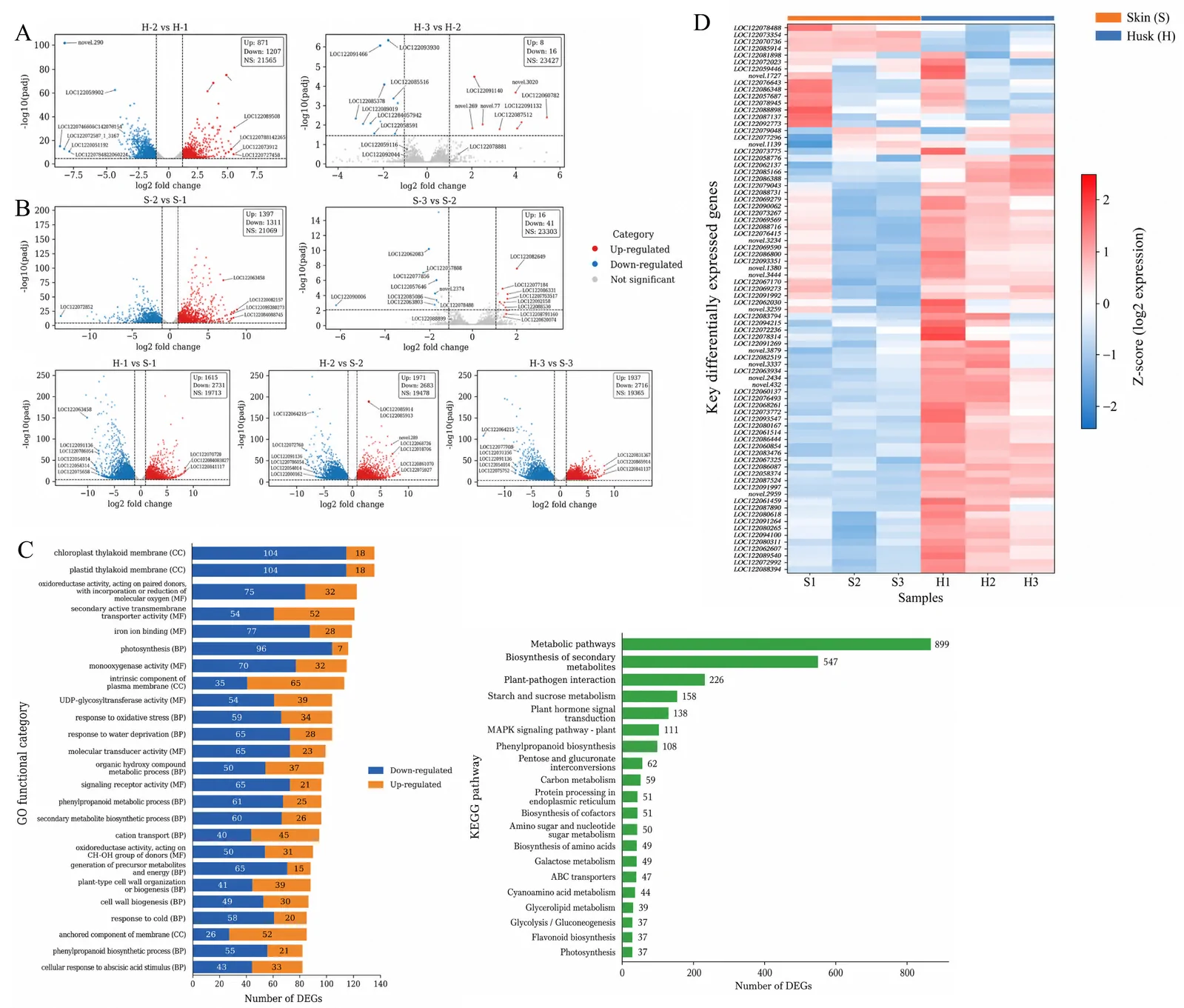
Differential gene expression analysis during husk and shell development. (**A**) Volcano plots showing stage-wise comparisons within husk (H-2 vs. H-1, H-3 vs. H-2) and shell (S-2 vs. S-1, S-3 vs. S-2) samples. (**B**) Volcano plots showing comparisons between husk and shell samples at the same developmental stage (H-1 vs. S-1, H-2 vs. S-2, H-3 vs. S-3). (**C**) GO functional classification and KEGG pathway classification of differentially expressed genes identified in the H-3 vs. S-3 comparison. (**D**) Heatmap showing the expression patterns of key differentially expressed genes in husk and shell samples. Each comparison included three biological replicates (*n* = 3). Differentially expressed genes were identified using DESeq2 with |log2FoldChange| ≥ 1 and adjusted *p* value (FDR) < 0.05. Expression values shown in the heatmap were generated from the normalized gene expression matrix.

To compare gene expression changes between husk and shell during development, differential expression analyses were performed using two types of pairwise contrasts: developmental stage comparisons within the same tissue and tissue comparisons between husk and shell at the same developmental stage (Figure 3). For within-tissue developmental comparisons, husk samples were compared as H-2 vs. H-1 and H-3 vs. H-2, whereas shell samples were compared as S-2 vs. S-1 and S-3 vs. S-2. In husk tissues, 871 up-regulated and 1207 down-regulated genes were identified in the H-2 vs. H-1 comparison, indicating a relatively large number of significantly differentially expressed genes at this transition. By contrast, only 8 up-regulated and 16 down-regulated genes were detected in H-3 vs. H-2 (Figure 3A). Similarly, in shell tissues, 1397 up-regulated and 1311 down-regulated genes were identified in S-2 vs. S-1, whereas only 16 up-regulated and 41 down-regulated genes were detected in S-3 vs. S-2 (Figure 3A). Thus, both husk and shell exhibited a similar pattern, with substantially more differentially expressed genes detected in the earlier developmental transition than in the later stage.

Comparisons between husk and shell at the same developmental stage revealed large numbers of differentially expressed genes at each corresponding stage (Figure 3B). Specifically, 1615 up-regulated and 1713 down-regulated genes were identified in H-1 vs. S-1, 1971 up-regulated and 2083 down-regulated genes in H-2 vs. S-2, and 1937 up-regulated and 1716 down-regulated genes in H-3 vs. S-3. All three stage-specific comparisons consistently showed high numbers of both up- and down-regulated genes, indicating that husk and shell maintained pronounced transcriptional differences throughout development.

Functional classification of the differentially expressed genes identified from the H-3 vs. S-3 comparison showed that they were mainly classified into the three major GO categories: cellular component, molecular function, and biological process (Figure 3C). Within these categories, multiple functional terms included both up- and down-regulated genes, suggesting substantial functional divergence between husk and shell at the mature stage across multiple biological dimensions. KEGG classification further showed that these differentially expressed genes were mainly involved in pathways such as metabolic pathways, biosynthesis of secondary metabolites, plant–pathogen interaction, and phenylpropanoid biosynthesis (Figure 3C). The heatmap of differentially expressed genes showed clear separation between husk samples (H-1, H-2, and H-3) and shell samples (S-1, S-2, and S-3) based on their expression patterns (Figure 3D). Some genes remained highly expressed in husk samples but showed low expression in shell samples, whereas others displayed the opposite pattern. Samples from the two tissues formed distinct clusters, further reflecting stable tissue-specific differences in gene expression between husk and shell.

### 3.4. GO/KEGG Enrichment Reveals Metabolic Pathways Associated with Shell Hardening

**Figure 4:**
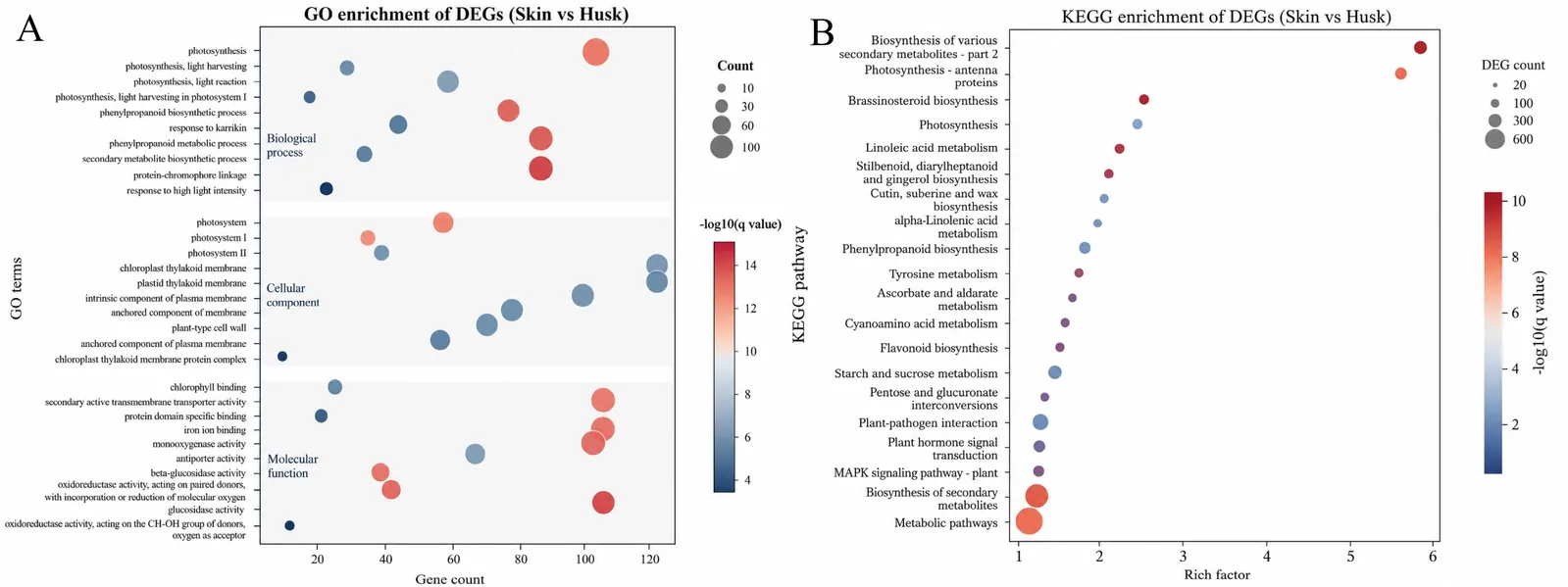
GO and KEGG enrichment analyses of differentially expressed genes between husk and shell. (**A**) GO enrichment bubble plot of differentially expressed genes between husk and shell, showing significantly enriched functional terms within the three major categories: biological process, cellular component, and molecular function. (**B**) KEGG enrichment bubble plot of differentially expressed genes between husk and shell. The x-axis represents the rich factor, bubble size indicates the number of differentially expressed genes in the corresponding pathway, and bubble color represents the significance level of enrichment. The complete set of expressed genes was used as the background gene set. GO terms and KEGG pathways with adjusted *p* value (FDR) < 0.05 were considered significantly enriched.

To further clarify the functional distribution of differentially expressed genes between husk and shell at the mature stage, GO and KEGG enrichment analyses were performed on the corresponding DEGs (Figure 4A,B). The GO results showed that shell-associated DEGs were mainly enriched in terms related to cell wall organization, lignin metabolic process, and oxidation–reduction process, whereas husk-associated DEGs were more strongly enriched in functions such as photosynthesis, chloroplast organization, and lipid metabolism (Figure 4A). Within the cellular component category, shell-associated DEGs were primarily distributed in the cell wall and extracellular region, while husk-associated DEGs were predominantly localized to structures such as chloroplasts and thylakoid membranes. KEGG enrichment analysis further indicated that these DEGs were mainly involved in metabolic pathways and the biosynthesis of secondary metabolites, with particularly strong enrichment in the phenylpropanoid biosynthesis pathway (Figure 4B). Within this pathway, several differentially expressed genes associated with lignin biosynthesis were identified, including genes involved in monolignol precursor formation and lignin polymerization. These genes included 4-coumarate–CoA ligase (4CL; LOC122058254), cinnamoyl-CoA reductase (CCR; LOC122058408 and LOC122060487), caffeoyl-CoA O-methyltransferase (CCoAOMT; LOC122092264), caffeic acid O-methyltransferase (COMT; LOC122057048, LOC122057467, and LOC122061057), caffeoyl shikimate esterase (CSE; LOC122057251), and multiple peroxidase genes (POD; LOC122057870, LOC122061145, LOC122061299, LOC122092439, and LOC122094301). These genes displayed differential expression patterns between husk and shell tissues, suggesting that they may contribute to lignin accumulation, secondary cell wall deposition, and cell wall reinforcement during shell development. In addition, pathways such as flavonoid biosynthesis, plant–pathogen interaction, plant hormone signal transduction, and the MAPK signaling pathway also included substantial numbers of DEGs. Taken together, these results suggest that the functional divergence between husk and shell at the mature stage is mainly reflected in cell wall-related metabolism, secondary metabolism, and signaling-related processes.

### 3.5. WGCNA Reveals Co-Expression Modules Associated with Mid-to-Late Shell Samples

**Figure 5:**
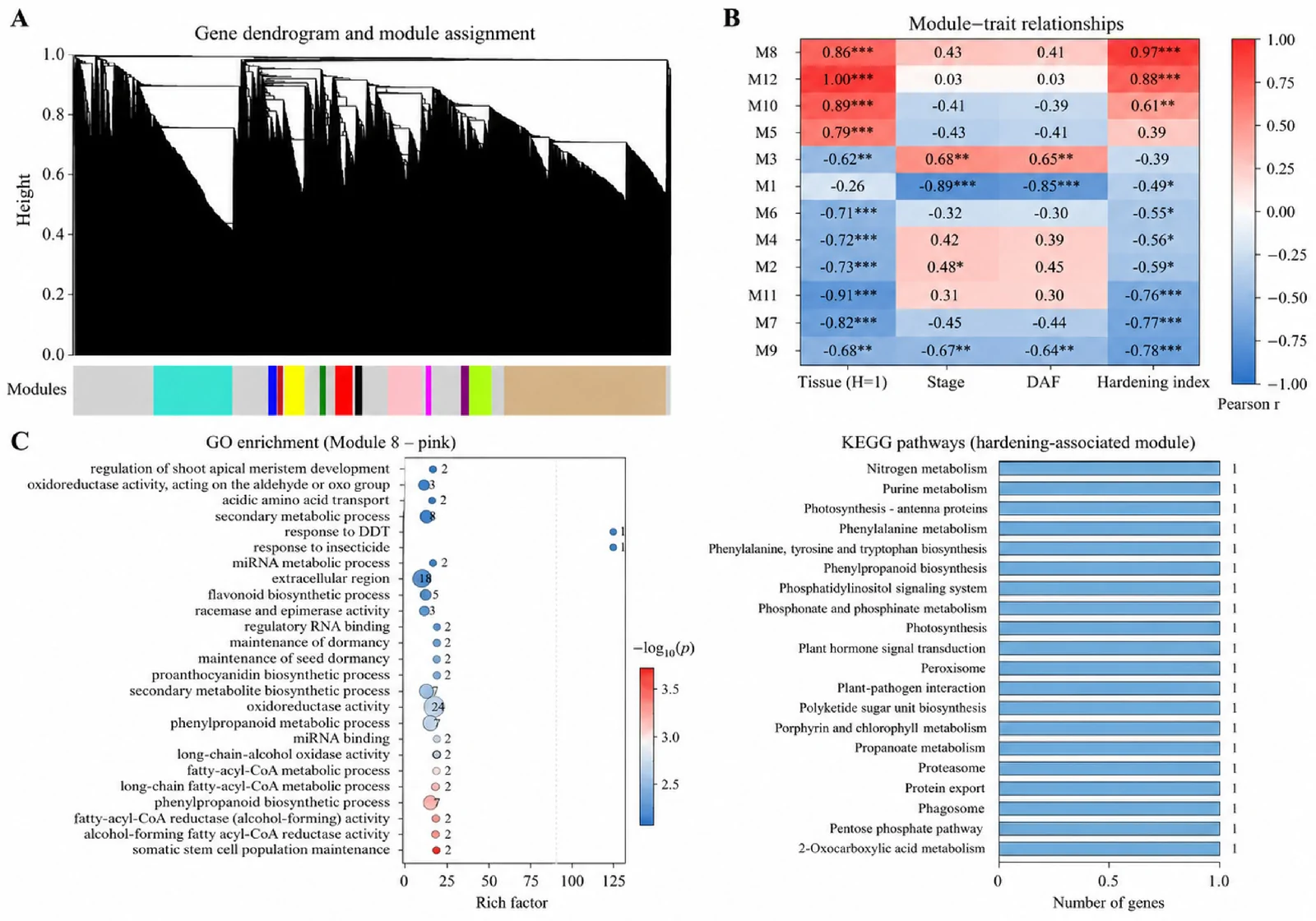
Co-expression network analysis and identification of modules associated with middle and late shell samples. (**A**) Hierarchical clustering dendrogram of genes and module assignment, with different colors representing distinct co-expression modules. (**B**) Heatmap showing the correlations between module eigengenes and sample traits, including tissue type, developmental stage, and days after flowering (DAF). Color indicates the Pearson correlation coefficient, and asterisks indicate statistically significant differences: * *p* < 0.05, ** *p* < 0.01, and *** *p* < 0.001, based on module–trait correlation analysis. (**C**) GO and KEGG enrichment results for the target module associated with middle and late shell samples. The left panel shows the GO enrichment bubble plot, and the right panel shows the KEGG pathway classification bar chart. Network construction was performed using the normalized gene expression matrix. Each developmental stage included three biological replicates (*n* = 3). GO terms and KEGG pathways with adjusted *p* value (FDR) < 0.05 were considered significantly enriched.

Hierarchical clustering and module detection results showed that genes with similar expression patterns were grouped into multiple co-expression modules, which were distinguished by different colors beneath the dendrogram (Figure 5A). Module size varied considerably, indicating differences in the extent of coordinated gene expression among gene sets across samples. Module–trait correlation analysis further revealed substantial variation in the strength of association between individual modules and sample traits, including tissue type, developmental stage, and days after flowering (DAF) (Figure 5B). Based on a comprehensive evaluation of these module–trait relationships, the M8 module showed a strong association with middle and late shell samples and was therefore selected as the target module for subsequent functional analysis (Figure 5B,C).

GO enrichment analysis showed that genes in the M8 module were mainly enriched in terms related to phenylpropanoid biosynthetic process, phenylpropanoid metabolic process, secondary metabolite biosynthetic process, flavonoid biosynthetic process, extracellular region, and oxidoreductase activity (Figure 5C). In addition, terms associated with long-chain fatty-acyl-CoA metabolic process, fatty-acyl-CoA reductase activity, and alcohol-forming fatty acyl-CoA reductase activity were also enriched. KEGG classification further indicated that genes in the M8 module were involved in multiple metabolism- and signaling-related pathways, including phenylpropanoid biosynthesis, phenylalanine metabolism, plant hormone signal transduction, plant–pathogen interaction, photosynthesis, and peroxisome (Figure 5C). Together, these results indicate that the target module is mainly composed of genes associated with secondary metabolism, redox regulation, and extracellular or structural functions.

### 3.6. Network-Based Prioritization of Candidate Hub Gene in the Module Associated with Mid-to-Late Shell Development

**Figure 6:**
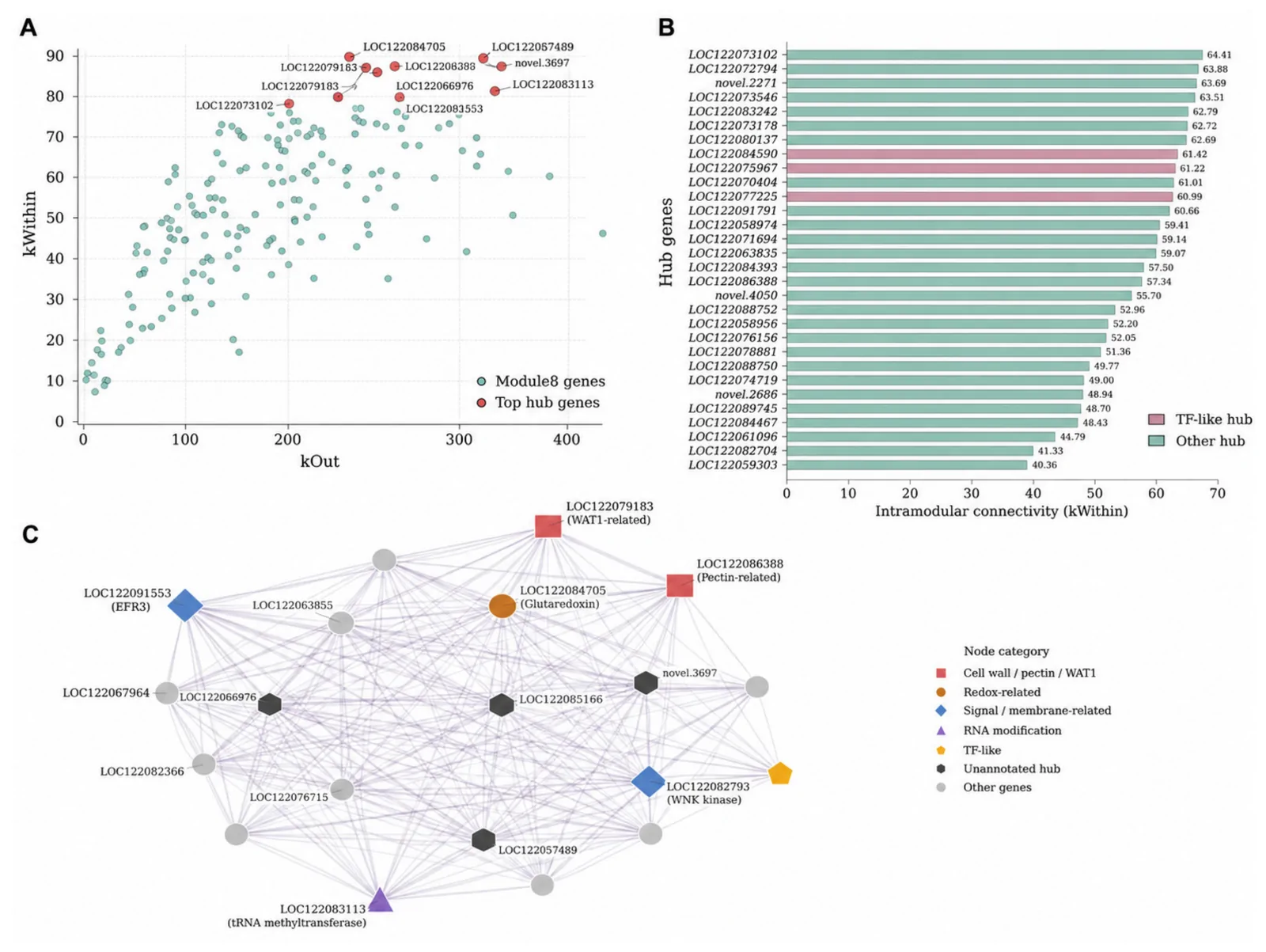
Candidate hub gene analysis in the module associated with middle and late shell development. (**A**) Scatter plot of intramodular connectivity (kWithin) versus extramodular connectivity (kOut) for genes in the target module. (**B**) Ranking of genes according to intramodular connectivity (kWithin). (**C**) Network graph of the target module showing the connection relationships among genes, with candidate hub genes highlighted. The co-expression network was constructed using the normalized gene expression matrix derived from three biological replicates (*n* = 3) for each developmental stage.

To further identify key node genes within the target module, the connectivity characteristics of genes in this module were statistically analyzed, and candidate hub genes were screened based on network topology (Figure 6). A scatter plot of intramodular connectivity (kWithin) versus extramodular connectivity (kOut) revealed clear differences in connection strength both within and outside the module among individual genes (Figure 6A). Notably, a subset of genes showed high kWithin values and was mainly distributed in the upper region of the scatter plot. By contrast, some genes exhibited relatively higher kOut values but lower kWithin values. Overall, candidate hub genes were identified based on their high network connectivity within the M8 module, rather than being considered experimentally validated rate-limiting regulators.

Based on the ranking of intramodular connectivity, a set of highly connected hub genes was identified (Figure 6B). The kWithin values varied substantially among genes, and the top-ranked genes showed stronger connectivity within the module, forming a core group of highly connected genes. Several of these genes were annotated as proteins associated with secondary metabolism, oxidation–reduction processes, or cell wall-related functions, suggesting potential roles in shell development. However, their precise biological functions in shell hardening require further experimental validation. The module network further illustrated the interaction relationships among genes within the target module (Figure 6C). High-connectivity candidate genes included those encoding a glutaredoxin family protein (LOC122084705), a WNK-type protein kinase (LOC122082793), a tRNA methyltransferase-related protein (LOC122083113), an EFR3 membrane-associated protein (LOC122091553), a pectin modification-related protein (LOC122086388), and a WAT1-related protein (LOC122079183). In addition, several genes without clear family annotation, including LOC122057489, novel.3697, LOC122085166, and LOC122066976, were also identified. These high-connectivity candidate genes in the target module represent functional categories related to redox regulation, signal transduction, RNA modification, membrane-associated processes, and cell wall modification.

### 3.7. Validation of RNA-seq Results by qRT-PCR

**Figure 7:**
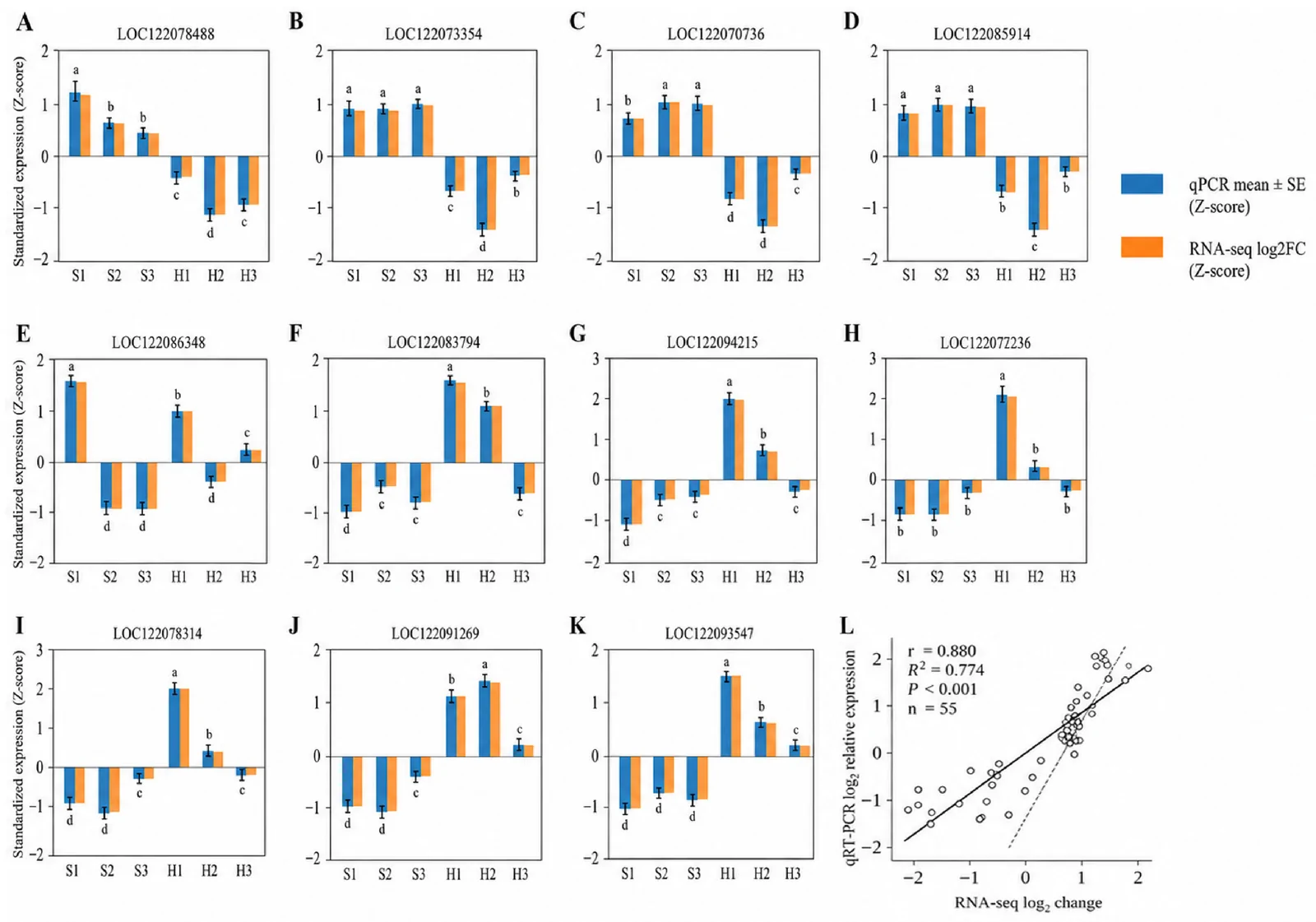
Validation of RNA-seq expression profiles of 11 representative genes by qRT-PCR. (**A**–**K**), Expression patterns of the 11 representative genes across six samples (S1, S2, S3, H1, H2, and H3) are shown, respectively. (**L**), Consistency analysis between RNA-seq and qRT-PCR expression results. The scatter plot illustrates the correlation between RNA-seq log_2_ expression changes and qRT-PCR log_2_ relative expression values based on 55 paired measurements. Pearson correlation analysis revealed a significant positive correlation between the two detection methods (r = 0.880, R^2^ = 0.774, *p* < 0.001), indicating the high reliability of the RNA-seq dataset. Different lowercase letters (a, b, c, and d) indicate significant differences among samples (*p* < 0.05).

To validate the reliability of the transcriptome sequencing results, the expression patterns of 11 candidate genes were examined by qRT-PCR (Figure 7). Overall, the expression profiles obtained by qRT-PCR were highly consistent with those generated by RNA-seq. The selected genes exhibited similar developmental expression trends across shell (S-1–S-3) and husk (H-1–H-3) tissues, although slight differences in expression magnitude were observed for some genes. Pearson correlation analysis further demonstrated a significant positive correlation between the RNA-seq and qRT-PCR datasets (r = 0.880, R^2^ = 0.774, *p* < 0.001, *n* = 55), indicating a high level of agreement between the two methods. These results confirm the reliability of the transcriptome sequencing data and support the validity of the subsequent differential expression and co-expression network analyses.

## 4. Discussion

The results of this study indicate that the critical transcriptional window for shell development in macadamia (*Macadamia* spp.) occurs between 30 and 50 days after flowering. During this period, the number of differentially expressed genes between adjacent developmental stages in both shell and husk tissues was substantially higher than that observed during the 50–80 DAF interval, when expression changes became markedly less pronounced. This pattern suggests that the core regulatory programs underlying late-stage tissue characteristics are largely established by the middle stage of development. Such a temporal pattern is consistent with developmental features reported in other highly lignified fruit tissues [5]. In peach (*Prunus persica*) [7], endocarp lignification occurs mainly during the early to middle stages of development, with genes involved in phenylpropanoid and lignin biosynthesis showing strong but transient induction, followed by a relatively stable maintenance phase. A similar pattern has been reported in walnut, in which the major lignification program of the endocarp is largely completed during the early to middle stages of fruit development, whereas subsequent development is mainly associated with structural maintenance [13]. However, comparisons of developmental timing among different species should be interpreted cautiously because developmental stages are defined using different criteria, including days after flowering (DAF), morphological characteristics, and anatomical changes. Therefore, the similarities observed between *M. integrifolia*, peach, and walnut likely reflect conserved regulatory processes associated with lignification and secondary cell wall formation rather than directly equivalent developmental time points. More broadly, transcriptomic studies of fruit development have shown that key structural formation is often concentrated within a transcriptional reprogramming window during mid-development [4]. In this respect, *M. integrifolia* resembles these species in that shell reinforcement is initiated not during late maturation, but earlier, during tissue differentiation and rapid wall formation. However, *M. integrifolia* also shows a distinct feature: although transcriptional changes between adjacent stages decrease at later developmental stages, the pronounced separation between husk and shell is maintained. This persistent divergence between tissues suggests that the late stage is not merely a static maturation phase, but rather a period during which the mechanical strength and barrier functions of the shell continue to be maintained [7,32]. Compared with previous transcriptomic studies in walnut and iron walnut, which mainly emphasized stage-specific activation of lignin biosynthesis and phenylpropanoid metabolism during shell formation [5,6,13], our results further demonstrate that distinct transcriptional programs between husk and shell are maintained throughout development, suggesting coordinated but tissue-specific regulatory mechanisms underlying shell hardening.

Husk and shell maintained pronounced expression differences across all three corresponding developmental stages. The clear separation observed in both the PCA and correlation heatmap further suggests that these two tissues enter distinct transcriptional trajectories early in development, rather than representing different degrees of differentiation along a single developmental path. At the mature stage, the comparison between husk samples (H-3) and shell samples (S-3) revealed pronounced tissue-specific functional divergence. Genes preferentially expressed in the shell were mainly associated with cell wall organization, lignin metabolism, extracellular-region functions, and redox-related processes, whereas genes preferentially expressed in the husk were more strongly associated with photosynthesis, chloroplast organization, and lipid metabolism. KEGG enrichment further demonstrated that phenylpropanoid biosynthesis and secondary metabolism were among the major pathways contributing to the transcriptional divergence between the two tissues. These findings are consistent with previous studies in walnut and other lignified fruit species, which have identified phenylpropanoid metabolism and lignin biosynthesis as conserved processes underlying shell hardening [5,6,13]. However, most previous studies focused primarily on differential gene expression or metabolic changes at individual developmental stages of *M. integrifolia* fruits [9,10]. In contrast, the present study combined multi-stage transcriptome profiling with WGCNA, allowing the identification of shell-associated co-expression modules and candidate hub genes that potentially coordinate shell development. This integrative strategy extends previous transcriptomic studies by providing a systems-level view of co-expression networks underlying shell hardening in *M. integrifolia*. Taken together, these results indicate that the observed tissue-specific expression pattern reflects not merely the activation of individual metabolic pathways, but a coordinated molecular program underlying functional specialization between the husk and shell [9,10]. Such inter-tissue differentiation is consistent with observations in fruits such as peach (*Prunus persica*) [7] and *Camellia chekiangoleosa* [33], in which endocarp or shell tissues preferentially undergo secondary cell wall deposition and lignification, whereas outer tissues retain stronger features related to assimilation, pigment accumulation, or active primary metabolism. In *M. integrifolia*, this division of labor remains highly pronounced even at the mature stage. This sustained divergence is likely related to the greater mechanical strength required of the *M. integrifolia* shell, its more complex shell-layer microstructure, and the need to preserve long-term protective functions during late fruit maturation, thereby preventing attenuation of the structural boundary between tissues as ripening proceeds [34].

GO/KEGG enrichment analysis and WGCNA further integrated this process into a coordinated network involving phenylpropanoid metabolism, secondary metabolism, cell wall organization, extracellular region functions, and redox-related processes. The persistent enrichment of phenylpropanoid biosynthesis suggests that the supply of lignin precursors occupies a central position in shell development, consistent with previous studies on endocarp lignification in walnut (*Juglans regia* L.) [5] and stone cell lignification in *Camellia oleifera* fruits [35]. Lignin accumulation is therefore not an isolated event, but part of a structural assembly process that proceeds in coordination with cellulose and hemicellulose deposition as well as upstream transcriptional regulation [36]. The M8 module was significantly associated with middle and late shell samples and was enriched in phenylpropanoid biosynthesis, secondary metabolite biosynthetic processes, extracellular region functions, and oxidoreductase activity. Candidate hub genes identified within this module were not restricted to typical lignin structural enzymes, but also included genes encoding a WAT1-related protein, a pectin modification-related protein, a glutaredoxin family protein, a WNK-type protein kinase, and an EFR3 membrane-associated protein. These findings suggest that middle-to-late shell development represents a coordinated co-expression program initiated during mid-development and maintained into later stages, rather than a simple accumulation of scattered differentially expressed genes [36]. Similar modular characteristics have also been reported in endocarp studies of *Juglans sigillata* [6], in which lignin biosynthesis-related genes frequently form highly correlated co-expression modules together with upstream master regulators such as NAC and MYB transcription factors, structural enzyme-encoding genes, and genes involved in cell wall polymer biosynthesis, showing strong associations with specific developmental stages or tissue types [6,9].

## 5. Conclusions

Based on transcriptomic analyses of husk and shell development in macadamia (*Macadamia* spp.), this study systematically characterized the expression divergence between the two tissue types across different developmental stages and clarified the molecular basis associated with shell layer formation. The results showed that major transcriptomic reprogramming in both husk and shell occurred predominantly between 30 and 50 days after flowering, whereas the number of differentially expressed genes declined sharply during the 50–80 DAF interval. This finding indicates that the key molecular events underlying late-stage tissue differentiation and shell layer formation take place mainly during mid-development. Differential expression and enrichment analyses further showed that genes associated with shell development were mainly involved in phenylpropanoid metabolism, secondary metabolism, cell wall organization, extracellular region functions, redox-related processes, and plant hormone signal transduction. Weighted gene co-expression network analysis identified a co-expression module significantly associated with middle-to-late shell development, in which genes were predominantly enriched in phenylpropanoid metabolism, redox-related processes, and extracellular region functions. From this module, several high-connectivity candidate hub genes were identified, including those encoding a glutaredoxin family protein (LOC122084705), a WNK-type protein kinase (LOC122082793), a tRNA methyltransferase-related protein (LOC122083113), an EFR3 membrane-associated protein (LOC122091553), a pectin modification-related protein (LOC122086388), and a WAT1-related protein (LOC122079183). Collectively, these findings indicate that *M. integrifolia* shell formation is a coordinated process involving cell wall remodeling, secondary metabolism, redox homeostasis, and signaling regulation. By integrating temporal transcriptomic profiling with co-expression network analysis, this study identifies the key developmental stages and major molecular regulatory features of shell formation, thereby providing a theoretical basis for further elucidation of shell developmental mechanisms and for the functional validation of candidate genes.

## Figures and Tables

**Table 1:** Summary statistics of transcriptome sequencing output for 18 *Macadamia* samples.

Sample	Raw Reads	Clean Reads	Clean Base (G)	Error Rate (%)	Q20 (%)	Q30 (%)	GC Content (%)
H1	45273524	43166726	6.48	0.03	96.53	90.82	45.95
H1	46378960	44167962	6.63	0.03	96.37	90.53	45.65
H1	48231658	46233688	6.94	0.03	96.26	90.32	45.73
H2	46639118	43951840	6.59	0.03	96.44	90.67	45.78
H2	45776924	44172868	6.63	0.03	96.62	91.04	45.98
H2	51946332	49145138	7.37	0.03	96.53	90.8	45.84
H3	44380722	41609286	6.24	0.03	96.11	90.01	45.67
H3	45421714	41663538	6.25	0.03	96.38	90.5	45.95
H3	47277866	44091296	6.61	0.03	96.2	90.17	45.46
S1	45999152	44342896	6.65	0.03	97.17	92	45.75
S1	48603512	46949020	7.04	0.03	97.31	92.28	45.08
S1	51489662	50146852	7.52	0.03	97.22	92.14	45.11
S2	44576806	41974402	6.3	0.03	97.22	92.05	45.25
S2	55573878	53447898	8.02	0.03	97.13	91.89	45.06
S2	57817086	56259126	8.44	0.03	97.91	93.97	45.33
S3	48974172	46309382	6.95	0.03	97.44	92.72	45.19
S3	48709250	47218160	7.08	0.03	97.22	92.06	45.54
S3	51464264	49642782	7.45	0.03	96.71	90.93	45.3

## Data Availability

The raw RNA-seq sequencing data generated in this study have been deposited in the China National Center for Bioinformation under BioProject accession number PRJCA070568.

## Supplementary Materials

The following supporting information can be downloaded at: https://www.mdpi.com/article/10.3390/plants15162442/s1.
